# The Role of the Mediterranean Diet and Alcohol Consumption in Chronic Liver Disease Prevention: A Narrative Review

**DOI:** 10.3390/medicina61101777

**Published:** 2025-10-01

**Authors:** María Barbería-Latasa, Diego Martínez-Urbistondo, Miguel A. Martínez-González

**Affiliations:** 1 Department of Preventive Medicine and Public Health, Instituto de Investigación Sanitaria de Navarra (IdisNA), University of Navarra, 31008 Pamplona, Spain; mbarberia.3@unav.es; 2 Department of Internal Medicine, Area of Vascular Medicine-Madrid, Clínica Universidad de Navarra, 28027 Madrid, Spain; dmurbistondo@unav.es; 3 Biomedical Research Network Center for Pathophysiology of Obesity and Nutrition (CIBEROBN), Carlos III Health Institute, 28029 Madrid, Spain; 4 Department of Nutrition, Harvard TH Chan School of Public Health, Boston, MA 02115, USA

**Keywords:** Mediterranean diet, MASLD, alcohol consumption, metabolic dysfunction, liver disease

## Abstract

The traditional Mediterranean Diet (MedDiet) has consistently demonstrated robust benefits in reducing cardiovascular, metabolic, and oncologic risks. Its high content of anti-inflammatory and antioxidant compounds, particularly (poly)phenols, underscores why this dietary pattern has been extensively researched and widely adopted for managing various metabolic conditions. This article aims to conduct a narrative review of the association between the MedDiet (and its debated alcohol consumption pattern) and a reduced risk of liver disease, with a specific focus on the potential preventive role of the MedDiet on Metabolic Dysfunction-Associated Steatotic Liver Disease (MASLD), which is now the most prevalent chronic liver disease globally. To carry out this review, relevant articles were searched on PubMed and other databases. The evidence found contributed to identifying the gaps in knowledge and allowed for the main findings to be summarized. Available randomized controlled trials and prospective cohort studies consistently support the hypothesis that high adherence to the MedDiet effectively reduces hepatic fat content, improves liver enzyme levels, and mitigates fibrosis progression. Crucially, this dietary pattern simultaneously addresses the significantly high cardiovascular risk inherent in MASLD. Historically, low-to-moderate alcohol intake, particularly moderate red wine with meals, was assumed to be a beneficial component of the MedDiet. While some observational studies suggest potential cardiovascular benefits, implying a loss of some MedDiet benefits if alcohol is excluded, a growing body of evidence highlights a deleterious synergistic interaction between alcohol, visceral adiposity, hepatic steatosis, and metabolic dysfunction. Based on the available evidence, clinical guidelines recommend the MedDiet with exercise for the prevention and management of MASLD. However, the alcohol consumption in the Mediterranean is currently under strong controversy. Furthermore, recent guidelines now advise total abstinence in patients with advanced liver disease and caution even at earlier MASLD stages. Yet, these assertions are largely based on observational data, underscoring the need for large clinical trials to address this issue with first-level evidence.

## 1. Introduction

The Mediterranean Diet (MedDiet) is an ancestral dietary pattern that originates from the coastal regions of the Mediterranean Sea, such as Greece, Italy, and Spain. It is characterized by the consumption of fresh, seasonal foods, including olive oil as the main source of fat, fruit, vegetables, fish, and wine [1]. It gained prominence in the 1950s and 1960s through Ancel Keys’ landmark “Seven Countries Study” [2], which observed a lower incidence of cardiovascular disease (CVD) despite relatively large fat intake. This beneficial effect was primarily attributed to olive oil and its antioxidant properties [1].

Since then, the traditional MedDiet has become the most extensively studied diet worldwide, with substantial evidence demonstrating beneficial effects on cardiovascular health [3], diabetes [4], and cancer prevention [5]. Over the decades, a vast body of research, ranging from long-term observational studies to robust clinical trials and comprehensive meta-analyses, has consistently confirmed the MedDiet’s positive impact on overall health, increased longevity, and improved quality of life [6,7,8].

The association between MedDiet and CVD is probably the most extensively investigated topic. CVD is the leading cause of global mortality and significantly impairs quality of life, with its prevalence nearly doubling since 1990 [9,10]. This rise imposes a major burden of disability and premature death. Key risk factors include lifestyle choices like smoking, poor diet, and sedentarism, alongside crucial health metrics such as elevated glucose, LDL cholesterol, blood pressure, and BMI [11]. These risk factors contribute to the development of various metabolic diseases, including diabetes, obesity, metabolic syndrome, and dyslipidemia. Metabolic diseases are closely interconnected, and a sustained state of metabolic dysfunction over time significantly increases cardiovascular risk.

The liver is a key organ in metabolic homeostasis, and it is closely involved in the development of metabolic diseases. Therefore, liver injury or dysfunction is related to metabolic disorders. The most prevalent liver disease is metabolic dysfunction-associated steatotic liver disease (MASLD), affecting 30% of the global population and leading to the increasing chronic disease burdens [12,13]. MASLD, as its name suggests, is characterized by hepatic steatosis coupled with underlying metabolic dysfunction. If this steatosis persists, it can progress to steatohepatitis, an inflammatory state of the liver. In more advanced stages, chronic inflammation leads to hepatic fibrosis, ultimately culminating in cirrhosis, an irreversible scarring of the liver [14]. However, steatosis is a reversible condition with lifestyle changes, whereas cirrhosis is not. Therefore, early detection of MASLD and intervention in its initial stages will be paramount in a preventive approach [15,16]. The shift in terminology from non-alcoholic fatty liver disease (NAFLD) to MASLD underscored a fundamental aspect of this condition: metabolic dysfunction, while avoiding stigmatizing terminology. Concomitantly, its diagnostic criteria have evolved; it is no longer solely defined by steatosis and an alcohol intake of <20 g/day for women or <30 g/day for men, but rather by the presence of at least one of the following metabolic conditions: overweight or obesity, type 2 diabetes, dyslipidemia, or elevated blood pressure [14]. However, managing these patients is complex. The absence of effective pharmacotherapies, coupled with frequent multiple comorbidities, necessitates a holistic approach to their treatment [17]. This is why a 7–10% weight loss, achieved through dietary changes and physical activity, is recommended for patients with MASLD [18].

In this context, observational and clinical studies have demonstrated that the MedDiet reduces intrahepatic fat content, improves insulin sensitivity, and lowers circulating insulin levels [19,20,21]. Furthermore, there is robust evidence of significant improvements in CVD risk factors such as systolic blood pressure and triglycerides [21]. Additionally, the combination of the MedDiet with physical exercise has also proven effective for weight loss, which is recommended for MASLD patients. Indeed, a sedentary lifestyle is associated with a higher risk of MASLD [22,23]. Moreover, independently of weight loss, patients who engaged in physical exercise had lower intrahepatic fat content and an improved lipid profile [24,25,26].

Despite the known benefits of the MedDiet, red wine remains a point of controversy. Clinical guidelines include alcohol intake of 20–50 g/day in women and 30–60 g/day in men as criteria for separating MetALD from MASLD [27]. These alcohol intake levels overlap with the limits typically accepted for the definition of the MedDiet (10–50 g/day for men, 5–25 g/day for women) [28,29]. While some studies suggest wine may benefit MASLD [30,31], others question its role and argue that wine –and alcohol in general–, should be removed from the definition of the healthy MedDiet [32]. The ongoing UNATI trial aims to provide first-level evidence to clarify this globally relevant issue [3,32,33].

In light of the available evidence, the aim of this study was to conduct a narrative review and a synthesis of the available evidence on the role of the MedDiet in the prevention of chronic liver disease, with special consideration to the Mediterranean alcohol drinking pattern.

## 2. Mediterranean Diet and Global Metabolic Regulation

### 2.1. The Mediterranean Diet Pattern

The MedDiet is characterized by an abundant and varied consumption of fresh, seasonal foods. Extra virgin olive oil stands as the primary fat source, complemented by high consumption of seasonal and local fruits, vegetables, nuts, and legumes. This food pattern also emphasizes unrefined cereals and moderate amounts of fish and poultry while limiting red and processed meats, sweets, full dairy, and refined cereals. Another key component is the moderate intake of red wine consumed with meals [2,7]. However, the significance of the MedDiet lies not merely in its individual key nutrients, but rather in the synergistic interaction of these nutrients as a dietary pattern. This concept encompasses proportions, frequency, variety, and nutrient combinations, offering a more holistic and accurate understanding of diet’s effects on the human body compared to studying isolated nutrients [1,2,3,4,5,6,7,8].

**Figure 1:**
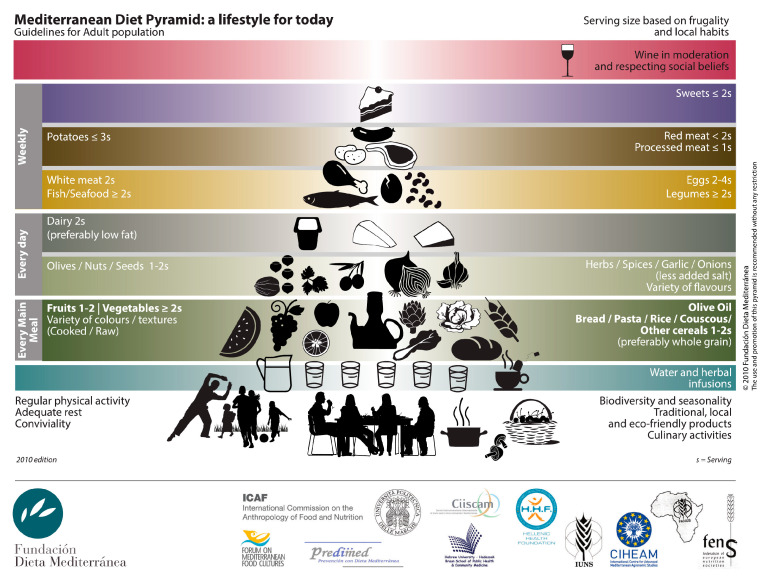
The Mediterranean diet pyramid.

As shown in Figure 1, the Mediterranean pattern, at its core, extends beyond a mere collection of elements like fruits, vegetables, olive oil, and cereals; it embodies a complete lifestyle. It encourages the consumption of traditional, locally sourced, environmentally friendly foods prepared using culinary techniques that enhance palatability. Furthermore, a fundamental aspect of this pattern is communal eating with family and friends, fostering social interaction around the table.

### 2.2. Mediterranean Diet and Health

In terms of macronutrient distribution, a notable feature of this pattern is its high fat content, often exceeding 40% of total caloric intake. First introduced to the scientific community in the 1960s by Ancel Keys, it was recognized as cardioprotective, despite its high fat intake contrasting with the low-fat diets recommended at the time. The primary source of this fat is olive oil, with a monounsaturated fatty acid profile known for its beneficial bioactive properties. This is particularly true for extra virgin olive oil (EVOO), which can account for more than 15% of the MedDiet’s total calories [1,34].

As previously mentioned, another key nutrient in this diet is the moderate consumption of red wine with meals, a distinguishing feature of the MedDiet compared to other healthy dietary patterns [4,7,32,35].

Mechanistic evidence supports the protective effects of the MedDiet, emphasizing the high biological plausibility of epidemiologic findings. The elevated consumption of fruits, vegetables, nuts, olive oil, red wine, and legumes, all rich in vitamins, phenolic compounds, and other antioxidants, confers a potent anti-inflammatory and antioxidant effect. This is extensively evidenced in relation to CVD and cancer [8]. Furthermore, a high intake of dietary fiber, capable of modulating the gut microbiota, is associated with a lower incidence of CVD and reduced blood pressure [36,37]. Another characteristic of this dietary pattern is its ability to mitigate the burden of cardiovascular disease risk factors [3,21]. This is achieved through the replacement of red and processed meats with fish and legumes, which provide healthier sources of proteins, a lipid profile richer in unsaturated fatty acids, and a greater contribution of fiber and vitamins. Similarly, another beneficial substitution in this diet is the replacement of dairy or sugary desserts with fresh fruit, as the habitual dessert, which provides higher contents of (poly)phenols and vitamins [8].

Beyond its extensively researched cardioprotective effects, the MedDiet has demonstrated protective effects against diabetes, obesity, and metabolic syndrome. These metabolic benefits, summarized in Table 1, are attributed to improved insulin sensitivity, a lower glycemic index, an enhanced lipid profile, and a reduction in oxidative stress (OS). Improved insulin sensitivity leads to better blood glucose control, with significant reductions in glycated hemoglobin (HbA1c) and fasting plasma glucose [37,38]. In these studies, insulin sensitivity was measured using a three-hour hyperinsulinaemic–euglycaemic clamp study. The incidence of diabetes was determined using an oral glucose tolerance test, and hepatic steatosis was assessed using localized magnetic resonance 1H spectroscopy. Furthermore, changes in the glycemic and lipid profiles were determined using fasting blood sampling. Plasma glucose concentrations were analyzed centrally using the glucose-oxidase method [21,36,37,38]. Moreover, although the MedDiet is not a calorie-restricted diet, its high fiber intake, emphasis on portion control, and consumption of unprocessed foods contribute to weight loss and maintenance, especially when combined with physical exercise in individuals with overweight and obesity [39].

Among the studies conducted on the MedDiet, randomized controlled trials (RCTs) warrant particular attention. The PREDIMED randomized trial evaluated the association of the MedDiet with the development of cardiovascular events in 7447 participants 50 years of age and older with high cardiovascular risk. Participants were randomized into three groups: MedDiet enriched with EVOO, MedDiet enriched with nuts, and a control group following a low-fat diet. After 4.8 years of follow-up, an approximate 30% reduction in the risk of cardiovascular events was observed in participants adhering to the MedDiet enriched with EVOO or with nuts, as compared to those assigned to receive advice on a low-fat diet. This RCT served as a cornerstone for dietary trials and globally demonstrated the cardioprotective power of the MedDiet [43]. Another trial that assessed the beneficial effects of the MedDiet on cardiovascular health was the secondary prevention trial CORDIOprev. This trial demonstrated that the MedDiet is not only effective for primary prevention but also reduced the relative risk of recurrent cardiovascular events by 26% after 7 years of intervention [44].

All these metabolic improvements directly influence the pathophysiological mechanisms involved in MASLD. The reduction in cardiovascular risk is particularly relevant for patients with MASLD, as CVD is the leading cause of morbidity and mortality in this population [45]. Similarly, the ability of the MedDiet to decrease insulin resistance, improve dyslipidemia, and exert anti-inflammatory and antioxidant effects is closely linked to the development and progression of MASLD [34,46].

## 3. Mediterranean Diet and MASLD

### 3.1. MASLD: Prevalence, Prevention, and Treatment

MASLD is the most prevalent chronic liver disease, affecting approximately 30% of the adult population worldwide. Its prevalence is projected to increase, partly due to its strong association with the global rise in obesity and diabetes [13]. Furthermore, MASLD is significantly linked to an elevated risk of cardiovascular disease, chronic kidney disease, and liver-related complications such as liver failure and hepatocellular carcinoma. Consequently, the burden of MASLD extends beyond the liver, posing a significant global health challenge with substantial socioeconomic implications [47]. MASLD has replaced the former term NAFLD and is characterized by excessive triglyceride accumulation in the liver, coupled with at least one cardiometabolic risk factor. MASLD encompasses various stages of liver damage: steatosis, steatohepatitis, fibrosis, and cirrhosis [47].

**Figure 2:**
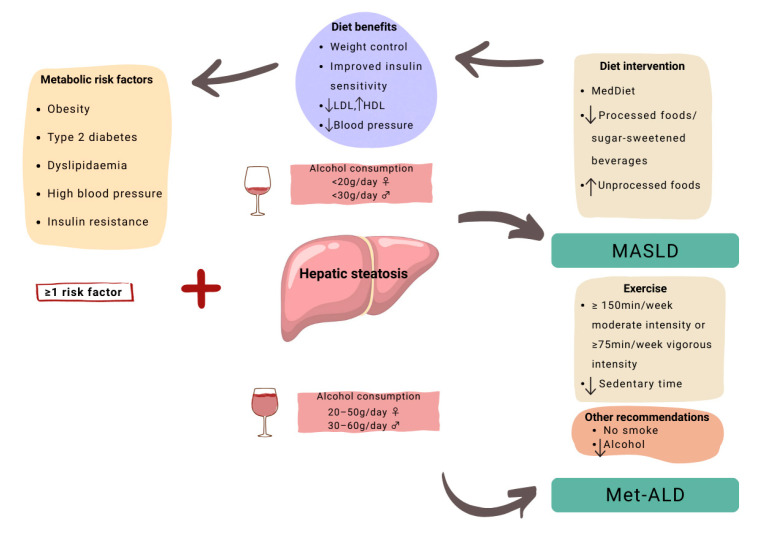
MASLD non-pharmacologic treatment.

The recommended non-pharmacological treatment for MASLD at any stage involves dietary modifications, increased physical activity, and lifestyle changes such as avoiding alcohol and tobacco. Regarding nutritional aspects, clinical guidelines primarily advocate for the MedDiet, along with reducing the consumption of processed foods and sugar-sweetened beverages (see Figure 2). This recommendation is based on the fact that several studies have found an association between processed food consumption and an increased incidence or progression of MASLD [48,49]. The efficacy of the MedDiet in managing MASLD is supported by numerous RCTs and observational studies that have consistently demonstrated its hepatic and cardiovascular benefits, even in the absence of weight loss [50,51,52].

### 3.2. MASLD and Mediterranean Diet: Intervention Trials

The high prevalence of cardiometabolic risk factors underscores the critical need for primary prevention of MASLD. The MedDiet has proven effective in controlling these risk factors and managing hepatic triglycerides. RCTs such as PREDIMED have demonstrated the benefits of the MedDiet on the Fatty Liver Index (FLI) in populations at high cardiovascular risk. Intervention groups consuming the MedDiet supplemented with EVOO or nuts showed a significant reduction in FLI compared to a low-fat diet group. These changes contribute to the delay or slowing of fatty liver progression, establishing the MedDiet as an effective therapy for preventing MASLD and halting its advancement [53,54]. Furthermore, a PREDIMED sub-study found a significant reduction in NAFLD prevalence in the group randomized to MedDiet and EVOO. After three years of follow-up, the prevalence of NAFLD in this group was significantly lower (8.8%) as compared to the control group (33.3%), highlighting the antioxidant and anti-inflammatory benefits of EVOO for liver health [55]. Another clinical trial, independent of PREDIMED, reported a significant 39% reduction in intrahepatic fat in participants treated with MedDiet-Green, a MedDiet enriched with phenolic compounds from an aquatic plant (*Wolffia globosa*) [40]. Once MASLD is diagnosed, the MedDiet has consistently demonstrated its effectiveness in several RCTs by improving metabolic parameters such as lipid profiles, glycemic indices, anthropometric measures, liver enzymes, and FLI [41,42,52].

### 3.3. MASLD and Mediterranean Diet: Observational Studies

Observational studies corroborate these findings. In the Framingham Third Generation cohort, higher adherence to the MedDiet was inversely associated with liver fat content, as measured by tomography. These changes were independent of glycemic control or changes in adiposity, suggesting mechanisms beyond weight loss, even in patients with a genetic predisposition to NAFLD [56,57]. Similarly, the CoLaus cohort study found an inverse association between MedDiet adherence and FLI [58]. Likewise, a cross-sectional study of overweight women found that high adherence to the MedDiet was associated with better FLI and hepatic steatosis index, regardless of menopausal status [59]. In line with these observations, studies conducted within the UK Biobank, a large prospective cohort including 500,000 individuals, confirm the inverse association between MedDiet scores and the risk of developing MASLD over 10 years of follow-up [60]. This cohort also revealed benefits of healthy diets, including the MedDiet, in the management of MASLD [61].

A compelling body of evidence underscores the usefulness of recommending the MedDiet as a dietary intervention for patients at cardiovascular risk to prevent MASLD and for those already diagnosed with MASLD to slow its progression. Despite the extensive evidence and endorsement by clinical guidelines for the use of the MedDiet in MASLD prevention and treatment, further efforts are needed to adapt it to Western countries. This requires tailoring the MedDiet to different cultural backgrounds and preferences, utilizing accessible materials such as videos, cookbooks, books, and webinars. Additionally, offering teleconsultations with nutritionists to MASLD patients could enhance long-term adherence to the MedDiet [17].

## 4. Alcohol Consumption and MASLD

### 4.1. Wine in the Mediterranean Diet

Wine (particularly red wine), alongside olive oil, is a defining component of the traditional MedDiet. The integration of wine into the MedDiet extends beyond a mere quantity of alcohol; it signifies a specific consumption pattern. This traditional pattern, prevalent in Mediterranean countries, involves the low-to-moderate intake of red wine consumed with meals [29]. Red wine distinguishes itself from other alcoholic beverages due to its high concentration of phenolic compounds, primarily found in grape skins. These compounds are largely responsible for the anti-inflammatory and antioxidant properties of red wine. Furthermore, red wine consumption has been associated with increases in HDL, improved insulin sensitivity, and enhanced platelet anti-aggregating capacity [62].

The Mediterranean pattern of alcohol consumption, as highlighted by Trichopoulou et al. [28], not only emphasizes red wine but also dictates the quantity and manner of its consumption. In 2014, Gea et al. developed the Mediterranean Alcohol Drinking Pattern (MADP) score to capture these nuances. This score considered moderate red wine consumption (5–25 g/day for women and 10–50 g/day for men) taken with meals, distributed throughout the week, while explicitly avoiding distilled spirits and binge drinking [29]. These seven aspects of the MADP characterize the typical alcohol consumption within the MedDiet and have been linked to reduced all-cause mortality, lower incidence of CVD, and decreased hypertension in various studies [32,63,64,65].

### 4.2. Alcohol, a Toxic Substance

Despite the observed benefits associated with this specific drinking pattern, it is crucial to acknowledge that alcohol is a toxic substance with well-established detrimental effects on health. The International Agency for Research on Cancer (IARC) classified alcohol as a Group 1 carcinogen. It is estimated that 4.1% of cancer cases can be attributed to alcohol consumption. Consequently, the World Health Organization (WHO) advocates for complete alcohol abstinence, asserting that no safe dose of consumption exists [32,63,66,67,68]. In the context of MASLD, and given the addictive properties of alcohol, the claim for total alcohol abstinence is supported by a variety of mechanisms, including interaction with cytochrome P450 2E1 (CYP2E1), the coupled effects of high ethanol intake and insulin resistance on enhancing OS. These mechanisms may promote lipid peroxidation and fibrogenesis. As alcohol intake increases, it contributes to micronutrient depletion with several metabolic and inflammatory derangements and also to sarcopenia and muscular impairment. Other mechanisms explaining its detrimental effects involve intestinal barrier disruption and mitochondrial dysfunction [69]. Consequently, it seems more prudent to discourage any alcohol intake in patients with MASLD. Nonetheless, contentious views on the overall effects of low-to-moderate alcohol intake, particularly on all-cause mortality in adult drinkers, persist [32,33,70,71]. Moreover, while numerous studies have linked alcohol, particularly red wine, to cardiovascular benefits, recent modeling, global assessments, and Mendelian Randomization (MR) studies now recommend alcohol abstinence for CVD prevention [63]. However, it is imperative to note that these studies often present limitations and methodologies that are not always verifiable, which can hinder their comparability or ability to substitute for an RCT [72,73]. Furthermore, a critical limitation of these studies is their inability to capture the intricate drinking pattern (including the different types of beverages), which likely acts as a significant effect modifier beyond the sheer quantity ingested [63].

### 4.3. Age-Stratified Recommendations

It is crucial to stratify and individualize the message regarding alcohol consumption. For younger individuals, alcohol use is often associated with binge drinking, which is linked to traffic accidents, injuries, psychiatric disorders, and suicides—major causes of death in this age group. In the 20- to 35-year-old population, where cardiovascular risk is typically low, no protective benefit from alcohol exists. In terms of liver disease, there has been an increase in the incidence of alcoholic liver disease among young people, particularly women, which leads to a high mortality risk in this group [74]. Therefore, for those under 35 years of age, total abstinence is unequivocally recommended [32].

However, for the population over 50 years old, two conflicting recommendations generate controversy: total abstinence or moderate alcohol consumption (≤14 drinks/week for men and ≤7 drinks/week for women) as a harm reduction strategy [71]. In this older demographic, particularly among those with high cardiovascular risk, adopting a Mediterranean drinking pattern may reduce the risk of CVD and premature death [3,32,63,70]. Consistent with this view, various studies on the MedDiet, which includes light-to-moderate red wine consumption with meals, have reported beneficial effects of this dietary pattern, including low-to-moderate alcohol intake on CVD, liver health, and other outcomes [43,51,75,76].

Furthermore, the role of red wine consumption within the MedDiet cannot be understated. In 2009, a study reported that removing the moderate alcohol consumption item from the Mediterranean Diet Score (MDS) resulted in a 23.5% loss of the MedDiet’s protection against all-cause mortality, making this alcohol consumption item the most relevant factor among nine items used to define the MedDiet [28].

### 4.4. Alcohol and MASLD

In MASLD patients, alcohol promotes hepatic steatosis, visceral adiposity, insulin resistance, inflammation, mitochondrial dysfunction, OS, and gut dysbiosis [45]. The combination of alcohol intake and central obesity strongly amplifies hepatic fibrogenesis, liver-related mortality, and cardiovascular complications. While minimal alcohol may be associated with some cardiovascular benefit in certain MASLD subgroups, consumption beyond 1–1.5 drinks per day clearly increases liver-related and global morbidity [45].

These data support the 2024 EASL-EASD-EASO guidelines recommending full alcohol abstinence in advanced MASLD and caution at earlier stages depending on comorbidity profiles [47].

It seems important to acknowledge that all observed effects on MASLD of low-to-moderate alcohol consumption are based solely on mechanistic studies with only intermediate end points or on observational studies, which may carry inherent biases. Common limitations of these studies include difficulties in establishing causal inference and biases such as the “sick-quitter effect” or “never starter sick” bias. These limitations compromise the validity of observational studies, especially if the reason for abstinence is a pre-existing illness or if the reported harms are only based on some of the intermediate postulated mechanisms without capturing a comprehensive picture, which will only be identified using final clinical end-points, such as all-cause mortality or hard CVD events. Consequently, an RCT involving drinkers over 50 years of age and using hard clinical endpoints is essential to resolve this ongoing controversy.

### 4.5. The UNATI Trial

**Figure 3:**
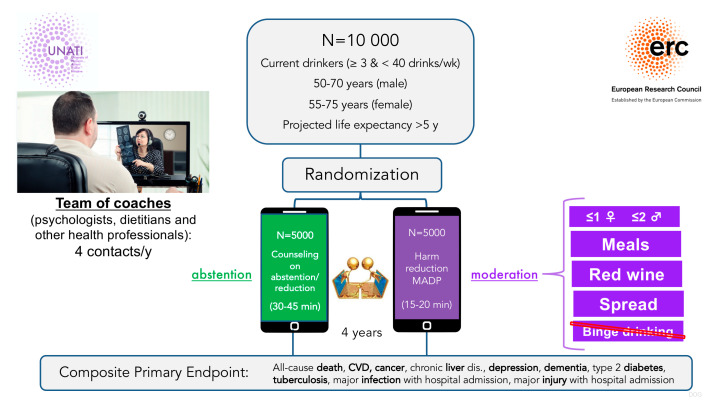
Randomization process for UNATI participants.

To resolve these controversies, the University of Navarra Alumni Trialist Initiative (UNATI), funded by the European Research Council, represents a landmark randomized controlled trial. Over 10,000 participants aged 50–75 years will be randomized to abstention or MADP interventions for 4 years (see Figure 3). Up to July 2025, already >8000 participants were recruited, and >6000 underwent randomization and intervention. UNATI will assess a composite endpoint including all-cause mortality, cardiovascular events, cancer incidence, injuries, and liver cirrhosis [32]. The nested UNATI-CUN substudy will incorporate imaging measures of hepatic fibrosis progression and coronary atherosclerosis, providing critical causal evidence on alcohol’s role in MASLD management.

## 5. Strengths and Limitations

This review summarizes the current evidence on the impact of the Mediterranean diet and alcohol consumption on liver disease, with a particular focus on MASLD. It also addresses the controversy surrounding the inclusion of alcohol as one of the items used to define the MedDiet. It summarizes the available observational evidence on the beneficial effects of moderate red wine consumption on cardiovascular health. However, it also acknowledges the limitations of these studies and emphasizes the need for large-scale randomized clinical trials, such as the UNATI trial, to guide future research and resolve the uncertainty surrounding the role of alcohol in liver and cardiovascular health. However, there are several limitations in our study that should be mentioned. Firstly, it is a narrative review rather than a systematic one. Secondly, much of the evidence concerning the relationship between alcohol and MASLD originates from observational studies, which may be subject to inherent shortcomings, particularly residual confounding, and cannot establish definitive causality. Finally, due to the lack of intervention trials, this review cannot offer a definitive conclusion on the exact role of alcohol in MASLD.

## 6. Conclusions

Future research should focus on defining individualized alcohol recommendations in patients with MASLD, balancing hepatic, cardiovascular, and oncologic risks. Large-scale randomized controlled trials such as UNATI and UNATI-CUN will provide crucial causal evidence to guide precision nutrition strategies.

The key findings of this article are as follows: (1) The MedDiet (whose operational definitions usually include moderate alcohol intake) has been shown to consistently reduce cardiovascular and metabolic risks, effectively lowering liver fat in patients with MASLD. Its high fiber content promotes weight loss and maintenance, especially when combined with exercise. (2) Moderate consumption of red wine with meals is a key feature of the MedDiet, which now seems to be highly controversial, particularly for patients with MASLD, and has been discouraged in many current clinical guidelines. Several studies have attributed anti-inflammatory properties and a protective effect against cardiovascular disease to red wine. However, much of the evidence on the role of alcohol comes from observational studies with inherent limitations. This fact highlights the need for large randomized controlled trials, such as the UNATI trial, to provide definitive causal evidence. (3) Until such evidence becomes available, the MedDiet remains the most important evidence-based dietary pattern associated with reduced metabolic and hepatic risks.

## Figures and Tables

**Table 1:** The main benefits of the MedDiet are to the metabolism.

Metabolic Condition	Benefits of the MedDiet	Key Studies
Insulin sensitivity	Improved insulin sensitivity; reduced fasting glucose and HbA1c.	[36,37,38]
Lipid profile	Reduced triglycerides and LDL-C; increased HDL-C.	[11,21]
Body weight	Contributes to weight loss and maintenance.	[39]
Inflammation and OS	Improved inflammatory markers and reduced OS.	[8,37,38]
Intrahepatic fat	Reduced intrahepatic fat content; improved hepatic biomarkers; prevention/slowing of progression.	[19,21,25,40,41,42]

HbA1c: glycated hemoglobin.

## Data Availability

Not applicable.

## Abbreviations

The following abbreviations are used in this manuscript:

MedDiet	Mediterranean diet
CVD	Cardiovascular disease
RCT	Randomized controlled trial
MDS	Mediterranean diet score
MR	Mendelian randomization
WHO	World Health Organization
IARC	International Agency for Research on Cancer
UNATI	University of Navarra Alumni Trialist Initiative
PREDIMED	PREvencion con Dieta MEDiterranea
OS	Oxidative stress
LDL	Low-density lipoprotein
HDL	High-density lipoprotein
BMI	Body mass index
NAFLD	Non alcoholic fatty liver disease
MASLD	Metabolic dysfunction-associated steatotic liver disease
EVOO	Extra virgin olive oil
HbA1c	Glycated hemoglobin
FLI	Fatty liver index
CYP2E1	Cytochrome P450 2E1
